# Harmonization of Flow Cytometric Minimal Residual Disease Assessment in Multiple Myeloma in Centers of Polish Myeloma Consortium

**DOI:** 10.3390/diagnostics11101872

**Published:** 2021-10-11

**Authors:** Agnieszka Krzywdzińska, Bartosz Puła, Anna Czyż, Beata Krzymieniewska, Jolanta Kiernicka-Parulska, Anna Mierzwa, Donata Szymczak, Aneta Milanowska, Aleksandra Kiraga, Iwona Kwiecień, Joanna Zaleska, Krzysztof Jamroziak

**Affiliations:** 1 Laboratory of Immunophenotyping, Institute of Hematology and Transfusion Medicine, 02-776 Warsaw, Poland; bkrzymieniewska@ihit.waw.pl; 2 Department of Hematology, Institute of Hematology and Transfusion Medicine, 02-776 Warsaw, Poland; bpula@ihit.waw.pl; 3 Department of Hematology and Bone Marrow Transplantation, Wroclaw Medical University, 50-367 Wroclaw, Poland; aczyz@onet.eu; 4 Flow Cytometry Laboratory, Haematology Clinical Laboratory, Department of Haematology and Bone Marrow Transplantation, University Hospital of Lord’s Transfiguration, 60-101 Poznan, Poland; jola@telvinet.pl (J.K.-P.); anna.m.mierzwa@gmail.com (A.M.); 5 Flow Cytometry and Cytomorphology Laboratory, Department and Clinic of Haematology, Blood Neoplasms and Bone Marrow Transplantation, University Hospital in Wroclaw, 50-367 Wroclaw, Poland; donata113@poczta.fm (D.S.); aneta.filipek94@gmail.com (A.M.); a.kiraga94@gmail.com (A.K.); 6 Laboratory of Hematology and Flow Cytometry, Department of Internal Medicine and Hematology, Military Institute of Medicine, 04-141 Warsaw, Poland; kwiecieniwi@gmail.com; 7 Department of Experimental Hematooncology, Medical University of Lublin, 20-059 Lublin, Poland; joannazaleska@umlub.pl; 8 Department of Hematology, Transplantation and Internal Medicine, Medical University of Warsaw, 02-097 Warsaw, Poland; kjamroziak@wum.edu.pl

**Keywords:** multiple myeloma, minimal residual disease, flow cytometry

## Abstract

Minimal residual disease (MRD) status is now considered as one of the most relevant prognostic factors in multiple myeloma (MM) while MRD negativity became an important endpoint in clinical trials. Here, we report the results of the first study evaluating the reproducibility of high-sensitivity flow cytometry MM MRD assessment in four laboratories in Poland. EuroFlow protocols for instrument setting standardization and sample preparation in MM MRD assessment were implemented in each laboratory. In the inter-laboratory reproducibility study, 12 bone marrow samples from MM patients were distributed and processed in participant laboratories. In the inter-operator concordance study, 13 raw data files from MM MRD measurements were analyzed by five independent operators. The inter-laboratory study showed high 95% overall concordance of results among laboratories. In the inter-operator study, 89% of MRD results reported were concordant, and the highest immunophenotype interpretation differences with regard to expression of CD27, CD45, CD81 were noticed. We confirmed the applicability and feasibility of the EuroFlow protocol as a highly sensitive method of MRD evaluation in MM. Results of our inter-center comparison study demonstrate that the standardization of MM MRD assessment protocols is highly desirable to improve quality and comparability of results within and between different clinical trials.

## 1. Introduction

Multiple myeloma (MM) is a hematopoietic neoplasm that remains incurable despite high rates of complete remission (CR) obtained with many novel chemotherapy or chemoimmunotherapy protocols [1]. New therapeutic options including new generations of immunomodulatory drugs and proteasome inhibitors as well as antibody-based and CART-cell targeted therapies, have enabled the achievement of even deeper responses in MM patients and significantly prolonged progression-free survival (PFS) and overall survival (OS) [2,3,4,5,6]. These advances have created the need for more sensitive diagnostic tools to detect residual tumor cells in the bone marrow (i.e., minimal/measurable residual disease, MRD), which is regarded as the major cause of relapse [7]. Therefore, increasingly sensitive assays such as multiparametric flow cytometry (MFC) and next-generation sequencing (NGS) have been adopted for the subclinical evaluation of bone marrow aspirates, providing brand new opportunities for both clinicians and patients [8].

Many studies, clinical trials and meta-analyses have confirmed the important prognostic role of MRD testing in MM patients. Regardless of the method used, achievement of MRD negativity is strongly associated with significant improvements in PFS and OS [9,10]. This association was seen in transplant-eligible and transplant-ineligible patients and those with newly diagnosed and relapsed or refractory disease. Furthermore, superior outcomes were observed in MRD-negative patients regardless of disease stage, cytogenetic risk or treatment received [11,12,13]. As a result, MRD status is considered as one of the most relevant prognostic factors in MM patients and MRD assessment is now one of the most active areas of research in MM [14]. As a surrogate endpoint in clinical trials, MRD status can accelerate drug development [15]. Furthermore, a result of the MRD assay could be potentially used as a prognostic factor for making treatment decisions and for informing timing of therapeutic interventions. Nevertheless, as the investigators highlight, standardization of MRD assays is highly needed to improve quality, comparability and interpretation of results within and between trials [16,17].

Based on current International Myeloma Working Group (IMWG) consensus of MM response criteria, MRD in bone marrow (BM) should be detected by sensitive, validated methods either by MFC, including next-generation flow cytometry (NGF) or by NGS, with a sensitivity level of at least 10^−5^ [18]. In an effort to decrease heterogeneity in approach and improve the accuracy of flow cytometry MM MRD testing, the International Clinical Cytometry Society (ICCS) and European Society for Clinical Cell Analysis (ESCCA) developed and published guidelines to harmonize antibodies panel, sample processing, analysis and results reporting [19,20,21]. In turn, the EuroFlow Consortium has pioneered a highly standardized NGF approach to monitor MRD in MM [22]. Under optimal test conditions, the MFC assay can reach sensitivity comparable to that achieved by the NGS method, and should allow us to detect one aberrant cell among 100,000 or even 1,000,000 normal cells (10^−5^–10^−6^) [22].

However, at least in Poland, a significant inter-center heterogeneity concerning MRD assessment in MM could be noted. In 2017, a survey study was conducted by the Polish Myeloma Consortium, with the aim to assess the technical capabilities and routine approach to MRD determination in patients with MM [23]. Although at that time, flow cytometry MM MRD assays were performed in only 46% out of the 15 surveyed clinical centers, all survey participants saw a need for establishing a routine MRD determination method in MM. Moreover, data received from seven flow cytometry laboratories revealed significant methodological discrepancies regarding MRD panel of antibodies construction (from three- to eight-color) and, even more importantly, the way of determining the sensitivity of the assays. The declared sensitivities differed 100-fold between laboratories (from 0.1 to 0.001%) with only two laboratories meeting all the methodological recommendations of ICCS/ESCCA. It is obvious that the MM MRD assay standardization is critical when test results would be used to inform clinical decisions. Therefore, in view of the planned clinical trials as well as to ensure good laboratory practice in routine clinical use, the Polish Myeloma Consortium attempted to disseminate MRD testing and promote standardization in Polish flow cytometry laboratories [24].

The aim of the present study was to assess the performance and comparability of results of MRD assessment in MM in four flow cytometry laboratories participating in clinical trials of the Polish Myeloma Consortium. We evaluated the inter-laboratory feasibility of standardization of flow cytometer settings and comparability of MRD results after implementation of EuroFlow procedures to local practice, as well as impact of expertise and operator interpretation on tests results.

## 2. Materials and Methods

### 2.1. Study Design

Four flow cytometry laboratories of Polish hemato-oncological centers were involved to the study, including: Flow Cytometry Laboratory of the Department of Hematology and Bone Marrow Transplantation, University Hospital of Lord’s Transfiguration in Poznan (further referred as Lab1); Flow Cytometry and Cytomorphology Laboratory, Department of Hematology, Blood Neoplasms and Bone Marrow Transplantation, University Hospital in Wroclaw (Lab2); Department of Experimental Hematooncology, Medical University of Lublin (Lab3) and as a Coordinating Laboratory, Laboratory of Immunophenotyping, Institute of Hematology and Transfusion Medicine in Warsaw (Lab4).

In 2019, an electronic survey was conducted, aimed at verifying compliance of the MRD assays protocols of the MM MRD assay in each laboratory. The participants were requested to provide categorized information regarding the MFC MRD assessment procedure including the type of instrument used, flow cytometer settings, antibody panels, staining procedure conditions, as well as the expertise of the staff in performing MRD tests in MM. The results of the survey were analyzed by the Coordinating Laboratory. Since all laboratories confirmed the use of the EuroFlow-adapted sample preparation protocol, in the first phase of our study, we decided to standardize instrument settings according to EuroFlow procedures. The required reagents and antibodies were acquired and distributed to the participants by the Coordinating Laboratory.

The second phase of the study aimed at assessing the inter-laboratory variability of myeloma PC measurements in the same BM samples, evaluated according to local protocols for MRD assessment in MM. In 2020, 12 BM samples (S1–S12), were prepared and distributed by the Coordinating Laboratory to the participating laboratories in three rounds. After evaluating the samples, the sites provided flow cytometry data files (fcs.) to the Coordinating Laboratory for analysis. Central analysis aimed also at determining the intra-assay variation (repeatability) and inter-laboratory comparison of the fluorescence intensity of the labeled antigens on normal plasma cells (PCs) obtained after instrument standardization.

The third phase of the study aimed at evaluating the inter-operator variability in MRD determination and MM plasma cell immunophenotype classification in the same cytometric data files. Raw cytometric data files (fcs.) of 13 patients with different MRD status (SA1–SA13) were electronically distributed to the participant laboratories by the Coordinating Laboratory.

After each study phase, the results of the comparisons were communicated to the participant laboratories and discussed.

### 2.2. Instruments Setup Standardization

Standardization of all flow cytometers settings was performed by implementation of the EuroFlow Standard Operating Protocol (SOP) for instrument setup and compensation for FASCCanto II and FACSLyric, respectively (www.euroflow.org, accessed on 7 October 2021) [25]. In order to setup photomultiplier (PMT) voltages in FACSCantoII instruments, we used median fluorescence intensity (MdFI) of the 7th reference peak of Rainbow beads calibration particles (Spherotech Inc., Lake Forest, IL, USA), EuroFlow-validated lot number EAK01. To set up standardized and comparable fluorescence measurements in FACSLyric flow cytometers, EuroFlow has defined specific tube target values (TTV) for each emission filter and fluorochrome. The appropriate tube settings and/or assays for FASCLyric are available on the EuroFlow website (www.euroflow.org, accessed on 7 October 2021). Before acquisition of the study samples, Rainbow beads of the same lot number were acquired, in order to monitor every instrument performance between study rounds. Moreover, participants were asked to acquire and record Rainbow beads on their routinely (i.e., before standardization) used cytometers settings.

### 2.3. Bone Marrow Samples

Bone marrow (BM) aspirates were collected from seven MM patients with variable tumor burden at routine response assessment visits (samples S1–S7). One sample (S4) was obtained from a patient after anti-CD38 therapy (daratumumab). Additionally, sample S7 was serially diluted in a remnant normal BM sample after immunophenotypic test collected from a patient without hematologic disease (S8–S12). All patients provided written informed consent according to the rules of the Institute of Hematology and Transfusion Medicine Ethical Committee (Protocol No. 14/2019, approval date 7 March 2019).

In total, 12 samples were distributed in three study rounds, including MRD-negative (*n* = 3) and MRD-positive (*n* = 9) at various levels (0.0018–5.9%) specimens, as assessed by the Coordinating Laboratory within 2 h after the draw (baseline MRD) (Supplementary Table S1). BM samples were collected in ethylenediamine tetra-acetic acid (EDTA) and did not include a stabilizing reagent. Anonymized samples were split equally and shipped by courier to the participating laboratories. To ensure similar measurement quality, BM samples in the Coordinating Laboratory were kept at room temperature for 24 h, and the assays were repeated simultaneously with the others participants.

### 2.4. Sample Preparation

Lyse–stain–wash method of sample preparation, as described in the EuroFlow protocol for NGF MRD assessment, was used (Supplementary Figure S1). Briefly, in the pre-lysis procedure, a high volume of BM sample was lysed before the cells were stained. It allowed for obtaining a sufficient number of leukocytes in a small sample volume. Two-tube 8-color panel of antibodies for PCs identification included: Tube 1—antibodies recognizing membrane antigens: CD27, CD138, CD38, CD56, CD45, CD19, CD117, CD81 and Tube 2—with the same antibodies as used in Tube 1, but instead of surface CD117 and CD81, intracellular anti-Kappa and anti-Lambda antibodies were included. Specific clones and supplier information can be found in Supplementary Table S2. After 15 min. incubation with antibodies against cell surface antigens, the cells in Tube 1 were lysed for the second time to eliminate residual erythrocytes and then washed. For intracellular light chain immunoglobulin detection in Tube 2, a fixative and permeabilizing reagent were used according to the manufacturer’s instruction. After washing in Phosphate Buffered Saline (PBS), the cells were resuspended and acquired on the flow cytometers as soon as possible after preparation. Current consensus guidelines require a minimum of 2 million and recommend 5 million events be acquired per tube for a sensitivity of 10^−5^ [22].

### 2.5. MM MRD Data Analysis

Central analysis of flow cytometry data files (fcs.) obtained during the inter-laboratory comparison sample preparation (S1–S12) was performed by the Coordinating Lab4 using analysis protocols in FACSDiva and FACSuite software for files from FACSCantoII and FASCLyric instruments, respectively. First, after cell doublets and debris exclusion, bone marrow nucleated cells population was defined. The total PCs population was identified by specific CD138, high CD38 and variable CD45 expression. Phenotypically aberrant PCs (aPCs) were identified by underexpression of CD19, CD27, CD38, CD45, CD81; overexpression of CD56; and asynchronous expression of CD117. A minimum of 2 aberrant phenotypes with light chain monoclonality (kappa or lambda) were required to define a cluster of clonal PCs. The number of viable nucleated cells was systematically registered, and the limit of detection (LOD) achieved in the assay was determined in each sample, according to the following formula: 20/number of nucleated cells × 100. Accordingly, the MRD was considered to be positive when a cluster of at least 20 aberrant cells was detected, and a sensitivity of 10^−5^ was obtained when at least 2 million nucleated cells, including erythroblasts, were collected.

### 2.6. Flow Cytometry Data Files

To evaluate the inter-operator variability in MM MRD results, electronic cytometric data files (fcs.) of 13 MM patients with different MRD level, were distributed by the Coordinating Laboratory. The files comprised: two MM MRD-negative cases, three with MRD detectable at level 10^−3^, three with MRD at 10^−4^ and five MRD at 10^−5^ level, including two cases after anti-CD38 therapy and one case with two subpopulations of neoplastic PCs. Laboratories were asked to analyze files using local software and gating protocols, and to report the number of pathological PCs as a percentage of nucleated bone marrow cells, along with their immunophenotype. At Lab4, fcs. files were analyzed independently by two cytometrists. In the immunophenotype concordance study, the results from individual operators were compared to the consensus immunophenotype defined for a given sample, which was the result reported by at least two operators.

### 2.7. Statistical Analysis

Analysis of intra-assays results, and fluorescence intensities study included determination of mean, standard deviations (SD), and percentage coefficient of variation (%CV). Excel 2016 (Microsoft Corporation, Redmont, WA, USA) and Graph Pad Prism v. 9.1 Windows (GraphPad Software, San Diego, CA, USA) were used for the graphical representation of data.

## 3. Results

### 3.1. MM MRD Assays Conditions in Study Participants

Participant laboratories had different experience in MRD assessment in MM—ranging from performing 50 tests per month in Lab1, to starting implementation of this assay in Lab3. All laboratories used the EuroFlow method of sample preparation which consisted of pre-lysis phase, however, various erythrocyte-lysing buffers were used. It should be noted that Lab3 changed the lysing reagent after one round of inter-laboratory variation study, from home-made ammonium-chloride based lysing reagent to BD PharmLyse (BD Biosciences, San Jose, CA, USA). The EuroFlow protocol includes a second short lysis step after antibody staining, and BD FACSLysing Solution (BD Biosciences, San Jose, CA, USA) with formaldehyde recommended for this step was used in two centers (Lab2, Lab3). In turn, BD PharmLyse not containing fixative agent was used in Lab4, whereas Lab1 skipped the second lysis step, by going directly to the washing step. Intracytoplasmic labeling for kappa and lambda light chain detection were performed by using two widely accepted permeabilization reagents.

To discriminate between phenotypically aberrant and normal plasma cells, all laboratories used a two-tube, eight-color antibody panel, recommended by EuroFlow. For patients after anti-CD38 immunotherapy, multiepitope CD38 antibody (CD38ME-FITC) was applied, instead of monoclonal CD38-FITC (Supplementary Table S2).

The Coordinating Laboratory (Lab4) used two flow cytometers FACSCantoII and FACSLyric (both manufactured by BD Biosciences, San Jose, CA, USA). In Lab1 and Lab2, samples were acquired in FACSCantoII, while in Lab3, in a FACSLyric instrument. Both types of flow cytometers used in the study were equipped with three lasers: blue (488 nm wavelength), red (633 nm for FACSCantoII and 640 nm for FACSLyric) and violet (405 nm). Cytometry data were analyzed with programs accompanying the instruments (BD FACSDiva or BD FACSuite) or third party software (Infinicyt, Cytognos, Salamanca, Spain). Only in one laboratory, Lab2, Infinicyt with Database was possible to use for analysis of MM MRD assays. Since the laboratories reported different instrument settings, we decided to implement the EuroFlow SOP for standardization of flow cytometer setup in each participating laboratory (Table 1).

### 3.2. Instruments Setup Standardization

The central analysis of the median fluorescence intensity (MdFI) values of Rainbow beads for each fluorescence detector, performed before settings standardization, showed differences between laboratories. A CV range of 9–48% for FACSCantoII and CV range: 9–65% for FACSLyric instruments were obtained, due to different local PMT voltage settings. After standardization of instruments’ settings, significant differences in MdFI were observed only in one study round and only for FACSCantoII cytometers (CV range 7–47%), when target MdFI values of the Rainbow beads were applied to the 8th peak instead of the 7th peak for instrument calibration in Lab1. In rounds 2 and 3, inter-laboratory comparability of signal achieved CVs below 10% for most fluorescence detectors in both types of cytometers (Supplementary Table S3).

### 3.3. Intra-Assay Variability (Repeatability)

Repeatability was determined in each laboratory and instrument (two for Lab4) by measuring the S1 BM sample five times in a single batch. Results of two levels of measurand were obtained: normal PC and neoplastic PC determination in two test tubes (with surface and surface/cytoplasmatic staining) were recorded and mean, SD and %CV were calculated. The results showed that satisfactory sample preparation precision was achieved in all laboratories (Supplementary Table S4). In Lab2 and Lab4, desirable %CV < 10 was reached in both staining tubes regardless of the PC level. Higher %CV values (here 21–23%) are acceptable, in samples with a low amount of the measurand. The experiment concluded that there was no significant intra-assay variation and it has provided a benchmark for comparing MM MRD assay performance in an inter-laboratory context.

### 3.4. Inter-Laboratory Variability Study

The distributed samples were received in approximately 24 h and cell staining took place at all laboratories 26–30 h after specimen collection. Central analyses were performed at the Coordinatig Laboratory by one operator, using analysis protocols in FACSDiva and FACSuite software for files from FACSCantoII and FASCLyric instruments, respectively. The inter-laboratory comparison study showed a high, 95% overall concordance of results among all laboratories and cytometers (Supplementary Table S5). During the first study round, an abnormally high amount of debris and non-lysed erythrocytes was found in specimens from Lab3 which resulted in a relatively low MRD result in S1 sample (0.74% vs. mean 1.30% in others laboratories) and lower LOD obtained in Lab3 for S2 sample (1.6 × 10^−4^ vs. mean 5 × 10^−6^ in other participants). With regard to this, it was decided to change the lysis reagent in Lab3 to BD PharmLyse buffer.

**Figure 1:**
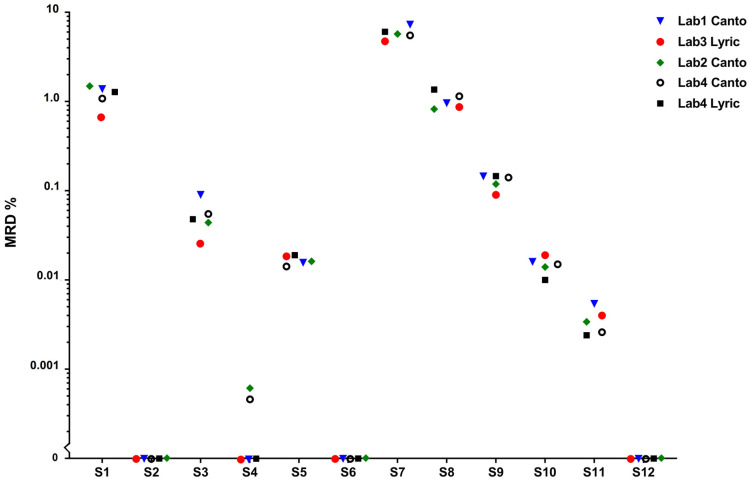
Results of MRD assessment in inter-laboratory comparability study. Samples 2, 6 and 12 did not contain pathological plasma cells.

In all three normal BM samples (S2, S6 and S12) aberrant PCs were not detected and a mean LOD of 1 × 10^−5^ (0.001%) obtained in the 15 negative determinations. The lower LOD (range 9 × 10^−6^—2 × 10^−6^) was reached in S12 due to an insufficient amount of distributed sample. In two samples with MRD at 10^−5^ level (S4 and S11) a mean LOD of 7 × 10^−6^ achieved. Nevertheless, only in Lab2 and Lab4CantoII it was possible to determine a cluster of cells which comprised >20 events required for MRD positivity (MRD result 0.0006% and 0.0008%, respectively). Thus, results of Lab1, Lab3 and Lab4Lyric were categorized as false negatives. In turn, all five measurements of sample S11 showed the presence of neoplastic plasma cell population with MRD range 0.002–0.005% (Figure 1).

Considering MdFI measurements of antigen expression on normal PC measured in three MM MRD samples, the results showed overall concordance between both types of cytometers after properly performed standardization. Erroneous instrument settings in Lab1 in round 1 resulted in lower MdFI values obtained for antigens but did not impact MRD detection in samples S1 and S2. For six out of 10 used markers, CVs of about 30% were achieved. The highest differences in intensity expression were detected in particular for CD138 (CV 65% for FACSCantoII and 92% for FACSLyric users), CD27 (CV 45 and 36%) and cytoplasmic kappa (CV 58 and 47%) and lambda (CV 55 and 45%) (Supplementary Table S6).

**Figure 2:**
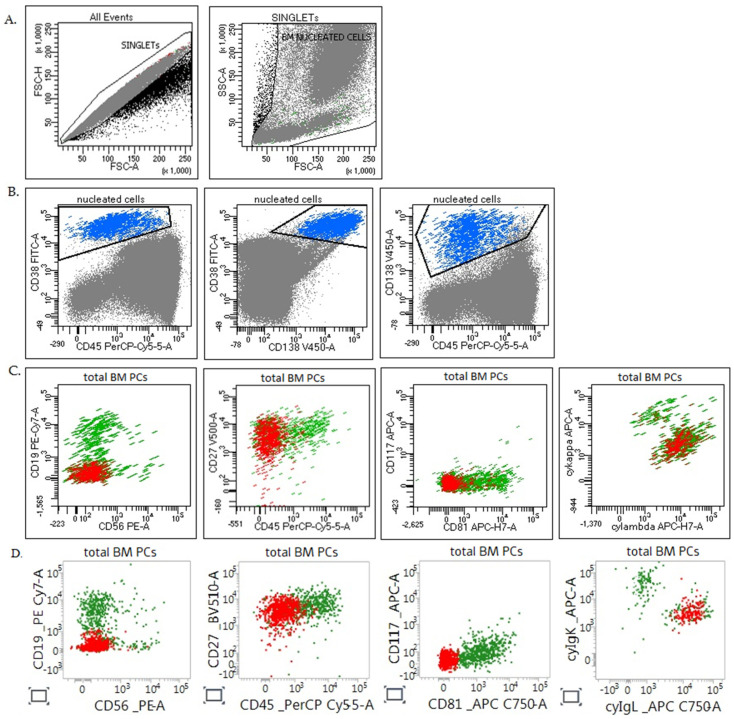
Gating strategy of MM MRD assessment in sample S9 (MRD = 0.13%). (**A**) Determination of nucleated cell population by excluding doublets (on FSC-Hight/FSC-Area dot plots) and cell debris (FSC-Area/SSC-Area); (**B**) Total PC population (blue dots) was determined by gating events CD38+ high CD138+ with variable expression of CD45. Neoplastic PCs (in red) were distinguished from normal PC (in green) by aberrant immunophenotype: CD19− CD56− CD27+ CD45− CD81− CD117− cytoplasmic lambda+; (**C**) Data from FACSCantoII Lab1; (**D**) Data from FACSLyric Lab3.

The gating strategy used for PCs identification in MM MRD assessment and an illustration of fluorescence obtained for the same sample in two cytometers is depicted in Figure 2.

### 3.5. Inter-Operator Variation Assessment (Fcs. Analysis)

To eliminate the sources of measurement variation resulting from transportation or sample preparation, 13 de-identified flow cytometry data files (fcs.) prepared in at the Coordinating Laboratory were sent for independent, blind analysis.

**Figure 3:**
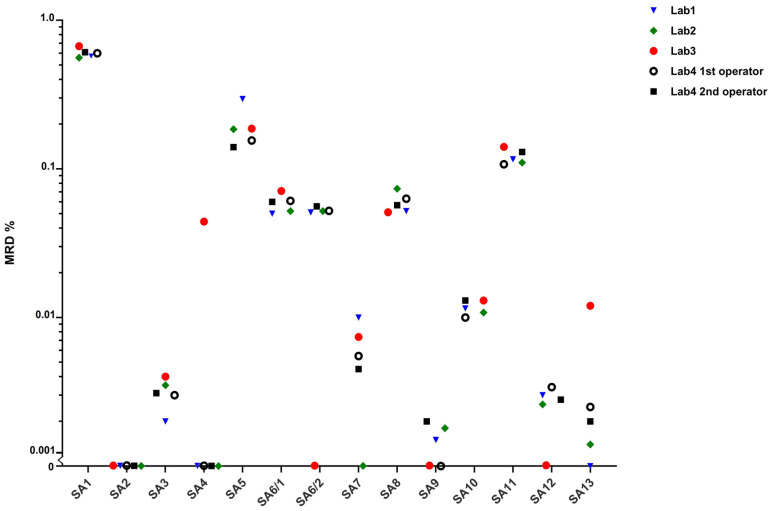
Results of inter-operator analysis variability study. Case SA6 with two different abnormal plasma cell populations, each accounted for >0.01% nuclear cells. Cases SA2 and SA4 did not contain pathological plasma cells.

In Lab1, Lab2 and Lab3 data analyses were performed with FACSDiva, Infinicyt with Database and FASCSuite software, respectively. In Lab4, files were analyzed by two operators using FACSDiva (1st operator) and Infinicyt software (2nd operator). Among 65 total MRD measurements in SA1–SA13 samples, the overall discordance rate was 11% and included six false negative and one false positive results (Supplementary Table S7). The full agreement was achieved for seven of 13 study cases (54%) (SA1–SA3, SA5, SA8, SA10, SA11). All operators detected the pathological PCs in all cases with MRD level of approximately 0.1% (10^−3^) and 0.01% (10^−4^), nevertheless the Lab3 result of SA6 was classified as a false negative, because only one of the two present aberrant PC subpopulations was identified. The consensus immunophenotypes of SA6 MRD populations were: aPC1 CD138+ CD38+ CD19− CD56+ CD27+ CD45+ CD117− CD81+ cylambda+ and aPC2: CD138+ CD38+ CD19− CD56− CD27+ CD45− CD117− CD81− cykappa+ and accounted for approximately 0.060% and 0.072% nuclear cells, respectively. As would be expected, the highest degree of inter-operator variation for samples with a very low (10^−5^) MRD level was recorded. Among five such samples, SA7, SA9, SA12, and SA13 were classified as false negative (Figure 3). More experienced operators from Lab1, Lab2 and Lab4 agreed on the presence or absence of MRD in 92–100% of study cases, nevertheless all but one of them made a mistake in MRD determination in cases with aPCs at the level of about 0.001%.

**Figure 4:**
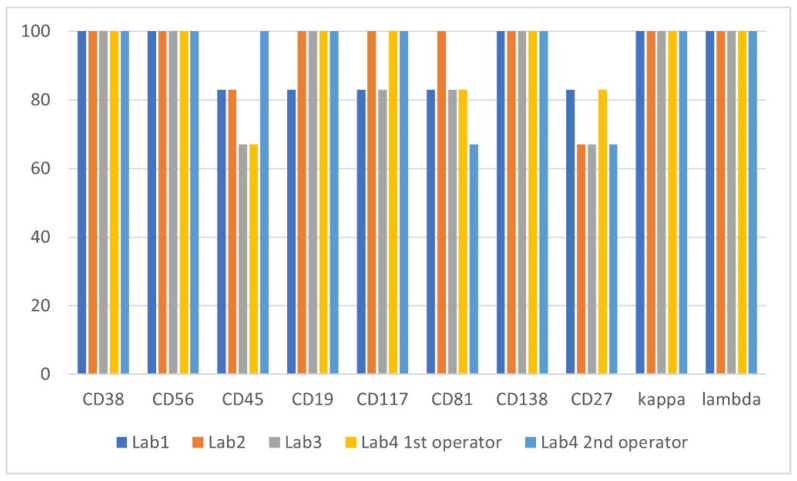
Concordance of antigen expression determination (in percentages) achieved for 6 study cases consistently identified as MRD-positive by 5 operators.

The inter-operator immunophenotype concordance study was assessed by comparing results of six cases that were correctly identified as MRD-positive by all operators (SA1, SA3, SA5, SA8, SA10, SA11). Antigen expression intensity on aberrant PCs was reported as positive, negative and dim or ± and consensus immunophenotype of MRD population for each case was defined as the result reported by minimum two operators. The highest variability was recorded for CD27, CD81 and CD45 antigen, with agreement of 73%, 80% and 83%, respectively (Figure 4). In one case, dim expression of CD19 on the tumor cells was not recognized, in two cases weak expression of CD117 was assessed as negative. The overall concordance of the MRD immunophenotype determination for each operator was 90%, 94%, 88% and 92% for Lab1, Lab2, Lab3 and both operators in Lab4, respectively.

## 4. Discussion

A significant number of anti-myeloma first-line combination therapies have been shown to result in a high response rate with over 50% patients achieving a response deeper than VGPR [18]. As a consequence, the implementation of MRD assessment gained importance due to its potential utilization of useful surrogate markers for patients’ response duration in clinical trials and routine practice. Indeed, studies have shown that MRD negativity is strongly correlated with prolonged patient PFS and OS [9,10]. Therefore, MRD negativity is often selected as the primary endpoint in clinical trials to assess the therapeutic potential of novel compounds that enable the shortening of follow-up or to test different response-adapted treatment strategies [26,27].

MFC is one of the techniques recommended by IMWG for the evaluation of MRD in MM [18]. The advantages of utilizing MFC for MRD assessment include broad availability, cost-effectiveness and fast turnaround time. However, as was also seen in our previous study, MFC MRD tests may suffer from interlaboratory variations caused by differences in antibody panel, staining protocols, methods of analysis and interpretation, and result in different sensitivity achieved [23,28,29]. Due to the relevance and usefulness of the MRD tests in MM, a series of efforts to standardize the technique have been undertaken. ICCS/ESCCA recommendations and NGF assays developed by the EuroFlow Consortium allow us to harmonize the approach to MRD testing. NGF is a two-tube, eight-color assay, with sample preparation protocols and Infinicyt software tools allowing us to reach the sensitivity of 2 × 10^−6^. The NGF assay was validated in several studies which confirmed its applicability, reliability and good correlation with NGS results [10,22,30]. Moreover, alternative approaches were initiated with the purpose of developing more cost-effective and easily usable MRD assays that offer the same performance [31,32]. It is worth noting that NGF can also be used to assess MRD in peripheral blood. Sanoja-Flores et al. showed that blood MRD-positive status in CR patients correlates with worse survival rates. Nevertheless, this method seems to be too insensitive to fully replace bone marrow assessment so far [33].

In our study, harmonization of the NGF MRD assessment method in MM was carried out in four clinical centers participating in PMC clinical trials. First, the NGF method of sample preparation for MM MRD assessment was implemented, albeit with differences in the erythrocyte lysis stage. Furthermore, the cytometer settings have been standardized according to EuroFlow SOPs for FACSCantoII and FACSLyric instruments. Harmonization of cytometer settings in laboratories participating in multi-center projects significantly facilitates the comparison of results and allows us to obtain high-quality data readings of fluorescence intensity, which in turn affects the correct interpretation of the measurement results [34]. In one of the first studies describing the interlaboratory standardization, Glier et al. showed the results of using the EuroFlow protocol in 10 cytometric laboratories in Switzerland [35]. The median fluorescence intensity (MdFI) of the applied markers was measured on the lymphocyte subpopulations in distributed blood samples, achieving a CV of <30% for seven out of used 11 markers with higher variability for CD38, CD56 and kappa/lambda measured on lymphocytes. In turn, after harmonization of FACSCanto and Navios (Beckman Coulter) instruments, Mathis et al. also obtained comparable results of MdFI measurements on patients’ PCs and high MRD correlation between flow cytometers for a cohort of 80 BM MM samples (*r*^2^ = 0.9798, *p* = 0.9621) [36]. Authors noted that the standardization of cytometer settings between laboratories may be crucial in particular for assessing samples with MRD percentage at the limit of the method sensitivity. In our study all participants managed to perform accurate setups, as reflected by the low interlaboratory variations (CV < 10%) of most fluorescence intensities measured on Rainbow beads. The concordance of cytometer settings was also verified by direct evaluation of MdFI on PCs in three samples, selected for this purpose as they contained only normal PCs. Our test sample was not large, but it showed a satisfactory agreement as measured by MdFI. The highest variations were noted for four markers: CD138, CD27 and cytoplasmic kappa and lambda light chains. They were visible independently of laboratory and type of cytometer, thus this seems to be related rather to sample transport conditions or individual biological variability of PCs. Weaker expression of CD138 is a common issue in long processed samples and might also be dependent on the lysing procedure [19,37]. In turn, insufficient washing of the plasma from BM samples in cyto-kappa and cyto-lambda staining might result in decreased labeling of immunoglobulin light chains. Staining of cytoplasmic markers requires accuracy and strict adherence to the procedures to avoid the observed differences in fluorescence measurements. The differences in expression of some markers found among participating laboratories did not compromise their ability to adequately determine PC population.

During the first round of inter-laboratory variability study, centralized analysis of the data obtained in individual laboratories allowed for the identification of quality issues caused by inappropriate lysis regent. The use of home-made lysing solution in Lab3 resulted in suboptimal staining with a low number of nuclear cells achieved. This resulted in too low MRD results in the sample S1 and low sensitivity obtained in sample S2 assessment. In contrast, analysis of results obtained during round 2 and 3 did not identify data quality issues that could be related to poor protocol adherence. It is worth noting that although the “pre-lysis” procedure is the recommended method to obtain a sufficient number of cells for staining, the other two methods: “pooled-tube” and “dextran sedimentation” were proposed for comparable BM population recovery rate and similar MRD detection sensitivity [37].

The goal of the inter-laboratory comparison phase of the study was aimed at determining the efficiency of the operating procedures to properly estimate MRD in the same bone marrow samples. Indeed, with the high 95% congruence of results observed, it was demonstrated that both standardization of instrument settings and harmonization of assay protocols allowed us to obtain reproducible MRD results. Nevertheless, we found we could not avoid issues with the quality of the distributed samples. This was seen in sample S4 where only in two of five cases a low number of clonal PCs, i.e., 0.0006% and 0.0008%, could be detected in central analysis (baseline MRD 0.002%). A 24 h cut-off from sample collection to staining is the standard in the context of multicenter clinical trials [20]. In our study, the discrepancies between results were observed in samples processed 24–28 h after the draw. The observed discrepancies were probably caused by decreased frequencies of fragile PC populations due to storage and transport conditions, although we must mention that cell viability was not determined.

The reliability of flow cytometric MM MRD testing is intrinsically related to the amount and the quality of the specimen. Inadequate handling and transportation, partially clotted and excessively hemodiluted specimens, are the major lab-independent errors we encounter. BM samples should be strictly insulated against excessive temperature changes during shipment and storage, and the tubes need to be labeled with the date and time of collection. The EuroFlow antibody panel does not include viability dye and the quality of the sample is assessed visually on the basic of FSC/SSC characteristics (Figure 2). Nevertheless, in the presence of apoptotic plasma cells (detected during analysis as decreased forward scatter and side light scatter) the viability should be assessed. Samples with <85% viability should be reported with a statement indicating that the viability is suboptimal for testing [20].

Highly representative and non-diluted BM samples are crucial for valid results of MRD evaluation and the first “pull” of aspirate is recommended [16]. Simultaneously, it is well-accepted that the percentage of PCs from bone marrow aspirate is usually under-represented. The focal nature of MM, hemodilution, and selective PC loss during processing are typically what most experts attribute to PC underestimation by MFC [38]. Importantly, MM MRD antibody combination enables the assessment of the overall quality of the BM aspirate through identification of BM-associated cell subsets, i.e., mast cells, hematogones, and erythroid precursors. Samples lacking these constituents are regarded as blood diluted and should be indicated as such on the final report [21,22].

The objective of harmonized practices in MFC is not only to issue the same tests results wherever samples are processed, but also to decrease the subjectivity of interpretation. The results of our inter-operator study showed satisfactory 89% overall agreement in the interpretation of results for the distributed 13 MRD cases. Nevertheless, it is worth noting that four out of five operators misinterpreted at least one MRD result. We identified seven discrepant results mainly in cases with very low MRD values (at 10^−5^ level). Correctly identifying residual disease in patients after anti-CD38 therapy (samples SA7 and SA9) was the most challenging. Importantly, according to EuroFlow recommendations, such cases require special multiepitope-CD38 antibody to circumvent antigen masking by daratumumab. This phenomenon may persist for 6 months after the end of treatment and makes gating and detection of PCs difficult, given reduced expression of CD38, the low number of pathological cells seen in the MRD setting and the instability of CD138 expression [39]. Another critical aspect is the knowledge of the immunophenotypic heterogeneity of normal PCs in bone marrow and the ability to distinguish even small differences in the expression of specific antigens, especially in cases when the baseline immunophenotype of MM cells is unknown. Moreover, it is important to note that the baseline tumour clone often shows also phenotypic heterogeneity, and all different phenotypic subclones should be followed throughout therapy [40].

Undoubtedly, reliability of the highly sensitive cytometric MRD examination strictly depends on the experience and knowledge of the operator, and the reduction of subjectivity in data analysis is an important advantage. The result of our study supports the findings from previous studies including the assessment of comparability of MRD tests results between the European Myeloma Network (EMN) flow cytometry laboratories [41]. In that study, 20 BM samples were tested using the standardized EuroFlow protocol and full concordance of MRD results was seen in 95% cases. Moreover, in the EMN study, qualitative expression of essential markers for PCs gating (i.e., CD38, CD138, CD45, CD19, CD56, cyto-kappa, cyto-lambda) showed a high degree of concordance between laboratories, whereas other markers (i.e., CD27, CD117, CD81) showed greater variability. In turn, in our study, we additionally compared the inter-operator concordance in the immunophenotype classification based on the same cytometric data. We noted the most common differences in determining expression were in CD27, CD81 and CD45 and were rather quantitative in nature, i.e., they concerned the marker expression differences classified as “+” vs. “dim or ±”. Altogether, the results of both studies underline the importance of using uniform analysis protocols, including recommendations for determining the phenotype of cells using internal positive and negative controls to reduce the subjectivity of data analysis [42].

We agree with Keeney et al. who in the summary of their study examining the reproducibility of MRD analysis results in patients with B-ALL in seven laboratories, stated that even experienced laboratories require constant monitoring and training [43]. They noticed that the discrepancies were, for the most part, due to errors in result interpretation, and the standardization of analysis protocols and educational workshops significantly increased the compliance between laboratories (discordance rate reduction from 26 to 9%) [43]. Therefore, in our opinion, highly sensitivity MRD assays should be performed by experienced laboratories, participating in external quality assurance programs or in interlaboratory comparisons. In multicenter studies, such as clinical trials, it is worth considering a preliminary study comparing laboratories, similar to the one presented in this article. Another option is the centralization of the data analysis, which in turn is facilitated by the standardization of the procedures of sample preparation and acquisition process. This seems to be an advisable solution for laboratories with lower experience in high-sensitivity MM MRD testing.

## 5. Conclusions

Our study proved the feasibility of EuroFlow protocols to ensure high reproducibility and efficient standardization of NGF MRD detection assays in MM in laboratories of Polish Myeloma Consortium centers. Most importantly, harmonization of MRD assays resulted in high concordance with regard to the presence or absence of MRD in inter-laboratory and inter-operator studies supporting the use of the method in multi-center clinical trials. Finally, the experience of operators is essential for a reliable interpretation of the results, especially in more demanding cases, such as patients after anti-CD38 therapy, with several subpopulations of pathological cells, or in cases with low MRD with the simultaneous presence of a highly heterogeneous population of normal BM PCs. In multicenter studies, such as clinical trials, it is worth considering a preliminary study comparing laboratories, similar to the one presented in this article.

## Figures and Tables

**Table 1:** Results of a preliminary survey and MM MRD assay conditions used in the inter-laboratory comparability study. Lab1—University Hospital, Poznan; Lab2—University Hospital, Wroclaw; Lab3—Medical University, Lublin; Lab4—Institute of Hematology and Transfusion Medicine, Warsaw.

	Lab1	Lab2	Lab3	Lab4—Coordinating Laboratory
**MM MRD tests per month**	50	<10	Start of MRD testing	<10
**Flow cytometer**	FACSCantoII	FACSCantoII	FACSLyric	FACSCantoII and FACSLyric
**Instrument settings** **before standardization**	Local setup	BD OneFlow Setup beads	Local setup	EuroFlow SOP
**Instrument settings** **after standardization**	EuroFlow SOP			
**Instrument QC**	BD CS&T beads according to manufacturer instructions			
**Analysis program**	FACSDiva	Infinicyt with Database	FACSuite	FACSDiva and Infinicyt
**Prelysis reagent**	BulkLysis Cytognos	BulkLysis Cytognos	Home-made NH_4_Cl in 1. study round; BD PharmLyse in 2. and 3. study round	BD PharmLyse
**Antibody panel**	Euro Flow 2 tubes 8-color			
**Assay protocol**	According to EuroFlow SOP			
**Lysis reagent after antibody incubation**	second lysis step not performed	BD FACSLysing Solution	BD FACSLysing Solution	BD PharmLyse
**Fixation/Permeabilization reagent for cytomplasmic antigens staining**	FIX&PERMCell Permeabilization Kit Invitrogen	IntraPrep Permeabilizaton Reagent BC	IntraPrep Permeabilizaton Reagent BC	FIX&PERM Cell Permeabilization Kit Invitrogen

QC—Quality Control; BD—Becton Dickinson Biosciences; BC—Beckman Coulter.

## Supplementary Materials

The following are available online at https://www.mdpi.com/article/10.3390/diagnostics11101872/s1. Table S1: Characteristics of samples distributed for the inter-laboratory comparison; Table S2: EuroFlow antibody panel used in PCM MRD assays. Multi-epitope CD38 antibody was used for patient after anti-CD38 therapy; Table S3. Standardization of flow cytometers settings and evaluation their stability using Rainbow beads calibration particles evaluated in three rounds of inter-laboratory comparison study; Table S4. Intra-assay variation (repeatability) results for two levels of measurand: high—for aberrant PC and low—for normal PC, assessed by testing S1 sample 5 times in a single analysis batch; Table S5. Concordance rates of MRD assessment in inter-laboratory comparability study; Table S6. Median fluorescence intensities (MdFI) comparison of antibodies used for detection normal PC population in bone marrow samples S2, S6, S12 of inter-laboratory comparison study; Table S7. Concordance rates of MRD assessment in inter-operator variability study; Figure S1. EuroFlow-NGF—based sample preparation protocol used in MM MRD studies.

## References

[1] Landgren O., Iskander K. (2017). Modern multiple myeloma therapy: Deep, sustained treatment response and good clinical outcomes. J. Intern. Med..

[2] Kumar S., Rajkumar S.V., Dispenzieri A., Lacy M.Q., Hayman S.R., Buadi F.K., Zeldenrust S.R., Dingli D., Russell S.J., Lust J.A. (2008). Improved survival in multiple myeloma and the impact of novel therapies. Blood.

[3] Durie B.G., Hoering A., Abidi M.H., Rajkumar S.V., Epstein J., Kahanic S.P., Thakuri M., Reu F., Reynolds C.M., Sexton R. (2017). Bortezomib with lenalidomide and dexamethasone versus lenalidomide and dexamethasone alone in patients with newly diagnosed myeloma without intent for immediate autologous stem-cell transplant (SWOG S0777): A randomised, open-label, phase 3 trial. Lancet.

[4] Lokhorst H.M., Plesner T., Laubach J.P., Nahi H., Gimsing P., Hansson M., Minnema M.C., Lassen U., Krejcik J., Palumbo A. (2015). Targeting CD38 with Daratumumab monotherapy in Multiple Myeloma. N. Engl. J. Med..

[5] Lonial S., Dimopoulos M., Palumbo A., White D., Grosicki S., Spicka I., Walter-Croneck A., Moreau P., Mateos M.V., Magen H. (2015). Elotuzumab therapy for relapsed or refractory Multiple Myeloma. N. Engl. J. Med..

[6] Teoh P.J., Chng W.J. (2021). CAR T-cell therapy in multiple myeloma: More room for improvement. Blood Cancer J..

[7] Paiva B., García-Sanz R., San Miguel J.F. (2016). Multiple myeloma minimal residual disease. Cancer Treat. Res..

[8] Oliva S., D’Agostino M., Boccadoro M., Larocca A. (2020). Clinical applications and future directions of minimal residual disease testing in multiple myeloma. Front. Oncol..

[9] Perrot A., Lauwers-Cances V., Corre J., Robillard N., Hulin C., Chretien M.L., Dejoie T., Maheo S., Stoppa A.M., Pegourie B. (2018). Minimal residual disease negativity using deep sequencing is a major prognostic factor in multiple myeloma. Blood.

[10] Paiva B., Puig N., Cedena M.T., Rosiñol L., Cordón L., Vidriales M.B., Burgos L., Flores-Montero J., Sanoja-Flores L., Lopez-Anglada L. (2020). Measurable residual disease by next-generation flow cytometry in multiple myeloma. J. Clin. Oncol..

[11] Munshi N.C., Avet-Loiseau H., Rawstron A.C., Owen R.G., Child J.A., Thakurta A., Sherrington P., Samur M.K., Georgieva A., Anderson K.C. (2017). Association of minimal residual disease with superior survival outcomes in patients with multiple myeloma: A meta-analysis. JAMA Oncol..

[12] Landgren O., Devlin S., Boulad M., Mailankody S. (2016). Role of MRD status in relation to clinical outcomes in newly diagnosed multiple myeloma patients: A meta-analysis. Bone Marrow Transplant..

[13] Munshi N.C., Avet-Loiseau H., Anderson K.C., Neri P., Paiva B., Samur M., Dimopoulos M., Kulakova M., Lam A., Hashim M. (2020). Large meta-analysis establishes the role of MRD negativity in long-term survival outcomes in multiple myeloma patients. Blood Adv..

[14] Kostopoulos I.V., Ntanasis-Stathopoulos I., Gavriatopoulou M., Tsitsilonis O.E., Terpos E. (2020). Minimal residual disease in multiple myeloma: Current landscape and future applications with immunotherapeutic approaches. Front. Oncol..

[15] Avet-Loiseau H., Ludwig H., Landgren O., Paiva B., Morris C., Yang H., Zhou K., Ro S., Mateos M.V. (2020). Minimal residual disease status as a surrogate endpoint for progression-free survival in newly diagnosed multiple myeloma studies: A meta-analysis. Clin. Lymphoma Myeloma Leuk..

[16] Costa L.J., Derman B.A., Bal S., Sidana S., Chhabra S., Silbermann R., Ye J.C., Cook G., Cornell R.F., Holstein S.A. (2021). International harmonization in performing and reporting minimal residual disease assessment in multiple myeloma trials. Leukemia.

[17] Rawstron A.C., Paiva B., Stetler-Stevenson M. (2016). Assessment of minimal residual disease in myeloma and the need for a consensus approach. Cytom. B Clin. Cytom..

[18] Kumar S., Paiva B., Anderson K.C., Durie B., Landgren O., Moreau P., Munshi N., Lonial S., Bladé J., Mateos M.V. (2016). International Myeloma Working Group consensus criteria for response and minimal residual disease assessment in multiple myeloma. Lancet Oncol..

[19] Flores-Montero J., de Tute R., Paiva B., Perez J.J., Böttcher S., Wind H., Sanoja L., Puig N., Lecrevisse Q., Vidriales M.B. (2016). Immunophenotype of normal vs. myeloma plasma cells: Toward antibody panel specifications for MRD detection in multiple myeloma. Cytom. B Clin. Cytom..

[20] Stetler-Stevenson M., Paiva B., Stoolman L., Lin P., Jorgensen J.L., Orfao A., Van Dongen J., Rawstron A.C. (2016). Consensus guidelines for myeloma minimal residual disease sample staining and data acquisition. Cytom. B Clin. Cytom..

[21] Arroz M., Came N., Lin P., Chen W., Yuan C., Lagoo A., Monreal M., de Tute R., Vergilio J.A., Rawstron A.C. (2016). Consensus guidelines on plasma cell myeloma minimal residual disease analysis and reporting. Cytom. B Clin. Cytom..

[22] Flores-Montero J., Sanoja-Flores L., Paiva B., Puig N., García-Sánchez O., Böttcher S., van der Velden V.H.J., Pérez-Morán J.J., Vidriales M.B., García-Sanz R. (2017). Next generation flow for highly sensitive and standardized detection of minimal residual disease in multiple myeloma. Leukemia.

[23] Krzywdzińska A., Solarska I., Puła B., Czyż A., Dytfeld D., Jurczyszyn A., Giannopoulos K., Balana-Nowak A., Własiuk P., Waszczuk-Gajda A. (2017). Praktyka kliniczna oceny minimalnej choroby resztkowej u chorych na szpiczaka plazmocytowego w Polsce: Badanie ankietowe Polskiego Konsorcjum Szpiczakowego. Hematologia.

[24] Jamroziak K., Krzywdzińska A., Solarska I., Puła B., Czyż A., Wróbel T., Giannopoulos K., Warzocha K., Dytfeld D. (2017). Znaczenie minimalnej choroby resztkowej w szpiczaku plazmocytowym—Stanowisko Polskiego Konsorcjum Szpiczakowego. Hematologia.

[25] Kalina T., Flores-Montero J., van der Velden V.H., Martin-Ayuso M., Bottcher S., Ritgen M., Almeida J., Lhermitte L., Asnafi V., Mendonca A. (2012). EuroFlow standardization of flow cytometer instrument settings and immunophenotyping protocols. Leukemia.

[26] Gozzetti A., Raspadori D., Bacchiarri F., Sicuranza A., Pacelli P., Ferrigno I., Tocci D., Bocchia M. (2020). Minimal residual disease in multiple myeloma: State of the art and applications in clinical practice. J. Pers. Med..

[27] Jamroziak K., Giannopoulos K., Wrobel T., Nowicki A., Pula B., Grzasko N., Rybka J., Robak P.J., Druzd-Sitek A., Walter-Croneck A. (2017). Preemptive Daratumumab Therapy for Minimal Residual Disease Reappearance or Biochemical Relapse in Multiple Myeloma: Rationale and Design of the Polish Myeloma Consortium Predator Study. Blood.

[28] Flanders A., Stetler-Stevenson M., Landgren O. (2013). Minimal residual disease testing in multiple myeloma by flow cytometry: Major heterogeneity. Blood.

[29] Keeney M., Halley J.G., Rhoads D.D., Ansari M.Q., Kussick S.J., Karlon W.J., Mehta K.U., Dorfman D.M., Linden M.A. (2015). Marked variability in reported minimal residual disease lower level of detection of 4 Hematolymphoid neoplasms: A survey of participants in the College of American Pathologists Flow Cytometry Proficiency Testing Program. Arch. Pathol. Lab. Med..

[30] Medina A., Puig N., Flores-Montero J., Jimenez C., Sarasquete M.E., Garcia-Alvarez M., Prieto-Conde I., Chillon C., Alcoceba M., Gutierrez N.C. (2020). Comparison of next-generation sequencing (NGS) and next-generation flow (NGF) for minimal residual disease (MRD) assessment in multiple myeloma. Blood Cancer J..

[31] Dold S.M., Riebl V., Wider D., Follo M., Pantic M., Ihorst G., Duyster J., Zeiser R., Wäsch R., Engelhardt M. (2020). Validated single-tube multiparameter flow cytometry approach for the assessment of minimal residual disease in multiple myeloma. Haematologica.

[32] Roshal M., Flores-Montero J.A., Gao Q., Koeber M., Wardrope J., Durie B.G.M., Dogan A., Orfao A., Landgren O. (2017). MRD detection in multiple myeloma: Comparison between MSKCC 10-color single-tube and EuroFlow 8-color 2-tube methods. Blood Adv..

[33] Sanoja-Flores L., Flores-Montero J., Puig N., Contreras-Sanfeliciano T., Pontes R., Corral-Mateos A., García-Sánchez O., Díez-Campelo M., Pessoa de Magalhães R.J., García-Martín L. (2019). Blood monitoring of circulating tumor plasma cells by next generation flow in multiple myeloma after therapy. Blood.

[34] Glier H., Novakova M., Te Marvelde J., Bijkerk A., Morf D., Thurner D., Rejlova K., Lange S., Finke J., van der Sluijs-Gelling A. (2019). Comments on EuroFlow standard operating procedures for instrument setup and compensation for BD FACS Canto II, Navios and BD FACS Lyric instruments. J. Immunol. Methods.

[35] Glier H., Heijnen I., Hauwel M., Dirks J., Ouarroz S., Lehmann T., Rovo A., Arn K., Matthes T., Hogan C. (2019). Standardization of 8-color flow cytometry across different flow cytometer instruments: A feasibility study in clinical laboratories in Switzerland. J. Immunol. Methods.

[36] Mathis S., Chapuis N., Borgeot J., Maynadié M., Fontenay M., Béné M.C., Guy J., Bardet V. (2015). Comparison of cross-platform flow cytometry minimal residual disease evaluation in multiple myeloma using a common antibody combination and analysis strategy. Cytom. B Clin. Cytom..

[37] Soh K.T., Tario J.D., Hahn T.E., Hillengass J., McCarthy P.L., Wallace P.K. (2020). Methodological considerations for the high sensitivity detection of multiple myeloma measurable residual disease. Cytom. B Clin. Cytom..

[38] Al-Quran S.Z., Yang L., Magill J.M., Braylan R.C., Douglas-Nikitin V.K. (2007). Assessment of bone marrow plasma cell infiltrates in multiple myeloma: The added value of CD138 immunohistochemistry. Hum. Pathol..

[39] Courville E.L., Yohe S., Shivers P., Linden M.A. (2020). VS38 Identifies myeloma cells with dim CD38 expression and plasma cells following daratumumab therapy, which interferes with CD38 Detection for 4 to 6 Months. Am. J. Clin. Pathol..

[40] Paíno T., Paiva B., Sayagués J.M., Mota I., Carvalheiro T., Corchete L.A., Aires-Mejía I., Pérez J.J., Sanchez M.L., Barcena P. (2015). Phenotypic identification of subclones in multiple myeloma with different chemoresistant, cytogenetic and clonogenic potential. Leukemia.

[41] Hofste op Bruinink D., Oliva S., Rihova L., Schmitz A., Gilestro M., Te Marvelde J., Kralova R., Høholt H., Broijl A., Johnsen H.E. (2020). Standardization of flow cytometric minimal residual disease assessment in international clinical trials—A feasibility study from the European Myeloma Network. Haematologica.

[42] Soh K.T., Wallace P.K. (2019). Monitoring of measurable residual disease in multiple myeloma by multiparametric flow cytometry. Curr. Protoc. Cytom..

[43] Keeney M., Wood B.L., Hedley B.D., DiGiuseppe J.A., Stetler-Stevenson M., Paietta E., Lozanski G., Seegmiller A.C., Greig B.W., Shaver A.C. (2018). A QA program for MRD testing demonstrates that systematic education can reduce discordance among experienced interpreters. Cytom. B Clin. Cytom..

