# Supplementary material for: Harmonization of Flow Cytometric Minimal Residual Disease Assessment in Multiple Myeloma in Centers of Polish Myeloma Consortium
Source: Diagnostics (Basel). 2021 Oct 11;11(10):1872. doi: 10.3390/diagnostics11101872 (PMC8534749; doi:10.3390/diagnostics11101872)
Supplement: Supplementary file 1 [file diagnostics-11-01872-s001.zip › diagnostics-1369010-supplementary.pdf]

Supplementary Materials

Supplementary Table S1. Characteristics of samples distributed for the inter-laboratory comparison.

Study Rounds	Sample Number	Baseline MRD%	Cells Count	Sample Volume/Lab	LOD Possible to Achieve
Round 1	S1	2.64	20 G/L	0.7 mL	6×10^{-6}
	S2	<LOD	50 G/L	0.5 mL	4×10^{-6}
Round 2	S3	0.060	10 G/L	0.5 mL	8×10^{-6}
	S4	0.002	180 G/L	0.5 mL	4×10^{-6}
	S5	0.028	30 G/L	0.5 mL	5×10^{-6}
	S6	<LOD	30 G/L	0.5 mL	5×10^{-6}
Round 3	S7	5.9	30 G/L	0.4 mL	6×10^{-6}
	S8*	1.77	13 G/L	0.3 mL	2×10^{-5}
	S9*	0.180	13 G/L	0.5 mL	1×10^{-5}
	S10*	0.022	12 G/L	0.8 mL	8×10^{-6}
	S11*	0.0018	12 G/L	2.0 mL	3×10^{-6}
	S12*	<LOD	12 G/L	0.8 mL	1×10^{-5}

* serial dilutions of MM patient bone marrow (S7) spiked in normal BM sample; MRD—minimal residual disease; LOD—limit of detection calculated as: 20/nucleated cells possible acquired from one tube ×100.

Supplementary Table S2. EuroFlow antibody panel used in MM MRD assays. Multi-epitope CD38 antibody was used for patient after anti-CD38 therapy.

Fluorochromes	BV510	BV421	FITC	PE	PerCP-Cy5.5	PE-Cy7	APC	APC-C750
**Tube 1**								
Antibodies	CD27	CD138	CD38	CD56	CD45	CD19	CD117	CD81
Clone	O323	MI15	CYT-38F or Multi-epitope CYT-38F2	C5.9	HI30	J3-119	104D2	M38
Company	BioLegend	BD	Cytognos	Cytognos	Cytognos	BC	BD	Cytognos
µL/Tube	10	2	6	2	5	5	5	6
**Tube 2**								
Antibodies	CD27	CD138	CD38	CD56	CD45	CD19	CyIgKappa	CyIgLambda
Clone	O323	MI15	CYT-38F or Multi-epitope CYT-38F2	C5.9	HI30	J3-119	Polyclonal	Polyclonal
Company	BioLegend	BD	Cytognos	Cytognos	Cytognos	BC	Dako	Cytognos
µL/Tube	10	2	6	2	5	5	5	3

APC: allophycocyanin; BV421: brilliant violet 421; BV510: brilliant violet 510; Cy: cytoplasmic; Cy5.5: cyanin5.5; Cy7: cyanin7; FITC: fluorescein isothiocyanate; Ig: immunoglobulin; PE: phycoerythrin; PerCP: peridinin–chlorophyll–protein. BC: Beckman Coulter; BD: Becton Dickinson Biosciences.

Supplementary Table S3. Standardization of flow cytometers settings and evaluation their stability using Rainbow beads calibration particles evaluated in three rounds of inter-laboratory comparison study.

	**MdFI%CV FACSCantoII (*n* = 3) before standardization**	**BD FACSCantoII * (*n* = 3)**						**MdFI %CV FACSLyric (*n* = 2) before standardization**	**BD FACSLyric ** (*n* = 2)**					
		**Round1**		**Round2**		**Round3**			**Round1**		**Round2**		**Round3**	
Fluorescence Detector	**%CV**	MdFI mean	**%CV**	MdFI mean	**%CV**	MdFI mean	**%CV**	**%CV**	MdFI mean	**%CV**	MdFI mean	**%CV**	MdFI mean	**%CV**
BV510	27	96 551	18	107 546	10	108 121	4	34	118 834	3	130 747	3	118 004	4
BV421	16	99 063	27	116 262	2	115 053	4	19	94 910	11	117 439	10	109 014	17
FITC	20	29 213	22	34 801	2	31 700	8	21	24 968	9	28 142	6	25 814	3
PE	36	32 050	33	38 913	3	37 010	5	38	39 316	9	40 266	5	40 037	3
PerCP-Cy5.5	9	61 693	47	78 090	2	74 781	6	11	104 258	5	105 128	6	102 711	3
PE-Cy7	24	10 218	7	10 683	11	10 253	8.	22	7 026	2	8 011	18	7 418	7
APC	19	153 139	28	180 623	3	176 779	5	9	146 793	3	156 873	6	150 362	2
APC-C750	48	72 080	9	74 211	4	72 460	6	65	52 929	3	59 172	7	52 185	2

* 7th reference peak of Rainbow beads (lot EAK01) was used to setup photomultipliers (PMT) voltages to reach the same MdFI in FACSCantoII cytometers according to EuroFlow procedure. ** To set up FACSLyric flow cytometers EuroFlow assay specific tube target values (TTV) available at www.euroflow.org, were implemented. MdFI—median fluorescence intensity; % CV—coefficient of variation expressed as a percentage.

Supplementary Table S4. Intra-assay variation (repeatability) results for two levels of measurand: high—for aberrant PC and low—for normal PC, assessed by testing S1 sample 5 times in a single analysis batch.

**Surface Staining**	**Aberrant PC**					**Normal PC**				
Instrument	FACSCantoII			FACSLyric		FACSCantoII			FACSLyric	
Participant	Lab1	Lab2	Lab4	Lab3	Lab4	Lab1	Lab2	Lab4	Lab3	Lab4
mean % (*n* = 5)	1.36	1.37	1.086	0.744	1.08	0.111	0.115	0.115	0.042	0.158
SD	0.195	0.094	0.027	0.121	0.053	0.023	0.004	0.012	0.003	0.008
%CV	14.4	6.3	2.4	16.3	4.9	21.1	3.9	10.5	7.0	4.8
**Surface/Cytoplasmic Staining**	Aberrant PC					Normal PC				
Instrument	FACSCantoII			FACSLyric		FACSCantoII			FACSLyric	
Participant	Lab1	Lab2	Lab4	Lab3	Lab4	Lab1	Lab2	Lab4	Lab3	Lab4
mean % (*n* = 5)	1.25	1.56	1.093	1.010	1.11	0.098	0.096	0.122	0.085	0.138
SD	0.085	0.026	0.035	0.053	0.036	0.015	0.007	0.009	0.016	0.005
%CV	6.7	1.7	3.2	5.3	3.2	14.8	6.8	7.5	18.8	3.3

%CV, coefficient of variation expressed as a percentage; SD, standard deviation

Supplementary Table S5. Concordance rates of MRD assessment in inter-laboratory comparability study. 12 distributed BM samples were tested for MM MRD in 4 laboratories on 5 cytometers. The data analysis was performed in the Coordinating Laboratory.

	**# Positive**	**# Negative**	**False Positive**	**False Pegative**	**Overall Concordance**
Lab1 FACSCanto II	8	4	0	1	92%
Lab2 FACSCanto II	9	3	0	0	100%
Lab3 FACSLyric	8	4	0	1	92%
Lab4 FACSCanto II	9	3	0	0	100%
Lab4 FACSLyric	9	3	0	1	92%

Supplementary Table S6. Median fluorescence intensities (MdFI) comparison of antibodies used for detection normal PC population in bone marrow samples S2, S6, S12 of inter-laboratory comparison study.

**Antibodies**	**FACSCanto (*n* = 3)**			**FACSLyric (*n* = 2)**		
	**MdFI mean (*n* = 9)**	**Range**	**%CV**	**MdFI mean (*n* = 6)**	**Range**	**%CV**
CD27 BV510	5.6	3.5–9.1	45	11.6	4.9–16.1	36
CD138 BV421	23.5	8.3–50.0	65	32.2	1.6–62.0	92
CD38 FITC	93.7	53.7–119.0	25	77.8	56.0–123.0	27
CD56 PE	0.36	0.2–0.5	26	0.3	0.2–0.4	20
CD45 PerCP-Cy5.5	9.5	4.6–14.0	31	13.8	9.3–18.3	30
CD19 PE-Cy7	13.0	7.0–18.3	24	9.0	5.3–11.9	27
CD117 APC	0.24	0.1–0.3	23	0.20	0.1–0.2	18
kappa APC	26.0	18.0–58.0	58	19.0	12.8–38.0	47
CD81 APC-C750	5.0	2.2–6.0	33	3.2	1.8–4.8	32
Lambda APC-C750	20.5	6.7–38.0	55	12.8	7.6–28.0	45

Supplementary Table S7. Concordance rates of MRD assessment in inter-operator variability study. 13 MM MRD flow cytometry files (fcs.) with varying levels of MRD were distributed and were analyzed in 4 laboratories by 5 independent operators.

	# **Positive**	# **Negative**	**False Positive**	**False Negative**	**Overall Concordance**
Lab1	10	3	0	1	92%
Lab2	10	3	0	1	92%
Lab3	9	4	1	3	69%
Lab4 1st operator	10	3	0	1	92%
Lab4 2nd operator	11	2	0	0	100%

Before starting protocol prepare the following:

a. Lysis buffer fixative—free (e.g. BulkLysis Cytognos, BD PharmLyse)

b. Lysis buffer with fixative (e.g. BD FACSLysing)

c. PBS (Phosphate Buffered Saline) with 0.2 %BSA

d. Antibodies used in 2 tubes 8-color MM MRD panel (specifications in Supplementary Table S2).

e. Fixation/permeabilization reagents kit for intracellular staining (e.g. FIX&PERM Cell Permeabilization Kit, Invitrogen; IntraPrep Permeabilizaton Reagent, Beckman Coulter

BULK-LYSIS PROCEDURE

- Assess sample cellularity
- Transfer at least 20×10^6 white blood cells to a 15mL conical tube.
- Add fixative—free lysis buffer in 1:9 proportion.
- Incubate at room temperature for 15min.
- Centrifuge at 800 ×*g* for 10min., discard the supernatant.
- Resuspend the cell pellet in 2 mL of PBS. Transfer to a 5mL FACS tube.
- Add 2mL of PBS, mix well.
- Centrifuge at 540 ×*g* for 5min, discard the supernatant.
- Adjust the final cells concentration to 1×10^5 cells/μL in volume of at least 300μL.

SURFACE STAINING
TUBE 1 and 2

- Transfer 100 to 200 μL of sample to two FACS tubes.
  Each tube should contain approximately 10×10^6 cells, otherwise use as much cells as it possible.
- Add antibodies for surface labeling to each tube. Mix well.

TUBE 1
SURFACE STAINING

- Add 2mL lysis buffer contained fixative.
- Incubate 10 min. at room temperature in the dark.
- Centrifuge at 540 ×*g* for 5 min., discard the supernatant.
- Add 2mL of PBS. Mix well.
- Centrifuge at 540 x*g* for 5min.
- Resuspend the cell pellet in 1mL of PBS. Mix well.

TUBE 2
INTRACELLULAR STAINING

- Add 2mL of PBS. Mix well.
- Centrifuge at 540 ×*g* for 5min.
- Resuspend cells in fixative reagent (medium A).
- Incubate 15min at room temperature in the dark.
- Add 2ml of PBS and centrifuge at 540 ×*g* for 5 min.
- Centrifuge at 540 ×*g* for 5 min., discard the supernatant.
- Resuspend cells in permeabilizing reagent (medium B).
- Add the intracellular antibodies. Mix well.
- Incubate 15 min at room temperature in the dark.
- Add 2mL of PBS. Mix well.
- Centrifuge at 540 ×*g* for 5 min.
- Resuspend the cell pellet in 1mL of PBS. Mix well.

ACQUISITION IN FLOW CYTOMETER

- Immediately after staining or (if not immediately acquired) store at 4 °C (for 1h maximum).
- In order to reach limit of detection and limit of quantitation at 10^{-5} level, recording of at least 2×10^6 and 5×10^6 nucleated cells for each tube is necessary.

Supplementary Figure S1. EuroFlow-NGF—based sample preparation protocol used in MM MRD studies.
